# The practice of lethal means restriction counseling in US emergency departments to reduce suicide risk: a systematic review of the literature

**DOI:** 10.1186/s40621-021-00347-5

**Published:** 2021-09-13

**Authors:** Amy A. Hunter, Susan DiVietro, Megan Boyer, Kristin Burnham, Danielle Chenard, Steven C. Rogers

**Affiliations:** 1 Department of Public Health Sciences, UCONN Health, 195 Farmington Ave, Suite 2100, Room U2012, Farmington, CT 06030 USA; 2 Injury Prevention Center, Connecticut Children’s and Hartford Hospital, Hartford, CT USA; 3 Department of Pediatrics, UCONN Health, Farmington, CT USA; 4 The Robert Larner College of Medicine, University of Vermont, Burlington, VT USA; 5 University of Connecticut-Storrs, Storrs, CT USA

**Keywords:** Suicide, Self-harm, Lethal means, Counseling, Restriction

## Abstract

**Background:**

Suicide is a leading cause of death in the US. Lethal means restriction (LMR), which encourages limiting access and reducing the lethality of particular methods of suicide, has been identified as a viable prevention strategy. For this approach to be successful, adequate education about risks and means must be communicated to families and individuals at risk for suicide. This systematic review aims to identify LMR methods most commonly communicated by healthcare providers in the emergency department, and barriers to the delivery of such counseling.

**Methods:**

The protocol for this systematic review is registered with PROSPERO (CRD42018076734). Included studies were identified through searching four databases (PubMed, Scopus, PsycInfo, and EBSCO). Studies were selected and coded independently by two researchers using the PICOS framework. Included studies examined LMR counseling delivered in the ED regardless of patient age, sex or race/ethnicity.

**Results:**

A total of 1282 studies were screened; 9 met the inclusion criteria. Included studies were published from 1998 to 2020. Study participants were majority female, and safe firearm storage was the most common form of LMR counseling provided. Eight studies included counseling on multiple forms of lethal means, [e.g., alcohol, medication, and firearm storage] and one study focused solely on safe firearm storage. Two studies reported barriers limiting healthcare providers’ delivery of LMR counseling, including lack of specialized skills and skepticism regarding the effectiveness of LMR counseling.

**Conclusion:**

There is limited published evidence that identifies the most effective methods and target populations for LMR counseling. Given the growing literature that provides evidence of gender differences in suicide modality (e.g., guns, medications, suffocation), LMR counseling should be multifaceted, to address common means of suicide in both men and women. Despite evidence that the majority of suicide attempts and half of completed suicides do not involve firearms, results showed that LMR counseling is frequently focused on promoting the safe storage of firearms. This highlights the need to include counseling focused on a variety of lethal means to reduce risk of suicide completion. Prospective studies should also aim to identify the most efficacious methods of delivering LMR counseling in the clinical settings.

## Background

Suicide is the second leading cause of death in individuals aged 10–34 years living in the United States (US) (Leading Causes of Death Reports, 1981–2018 2020). In 2018, approximately 48,000 deaths were attributed to suicide (14.8 per 100,000 population), which resulted in 1 death every 11 min (Leading Causes of Death Reports, 1981–2018 2020). Each year, suicidal behaviors result in $70 billion in lifetime medical and employment-related loss (Leading Causes of Death Reports, 1981–2018 2020). These financial costs, coupled with emotional devastation and the increasing rates of completed suicides clearly signal the need for an urgent response to this emerging public health epidemic.

Each year in the US over 500,000 people present to emergency departments (ED) for deliberate self-harm and/or suicidal ideation (Leading Causes of Death Reports, 1981–2018 2020), which are known risk factors for suicide (Ribeiro et al. 2016). It has been demonstrated that in the year following these visits, suicide mortality is 57-fold higher for patients who presented with deliberate self-harm and 31-fold higher among patients who presented with suicidal ideation (Goldman-Mellor et al. 2019), than in the general population. It is also estimated that over 12% of suicide fatalities involve individuals who were treated in an ED within three months of death (O'Neill et al. 2019), underscoring the importance of having effective prevention strategies available for ED providers and their patients. Indeed, the emergency department is an important point of contact between individuals at risk of suicide and medical professionals with the resources to conduct risk screening and timely intervention, such as providing preventive support, mental health referrals, and lethal means restriction (LMR) counseling. Understanding this, the Joint Commission enacted the Patient Safety Goal 15.01.01, EP6 which outlines requirements for counseling and follow up care at discharge for patients identified as at risk for suicide (Commission 2019).

LMR is defined as an approach to suicide prevention that reduces access to a fatal method of suicide (e.g. firearms, medications, sharps), thus preventing or reducing the lethality of an attempt. As such, LMR is considered a “program with evidence of effectiveness” by the national Suicide Prevention Resource Center. Yet, for this approach to be successful, counseling regarding suicide risk and methods of effective LMR must be communicated to individuals and families before the onset of suicidal crisis or subsequent attempts. Ideally, LMR counseling should be provided when suicide risk is first identified, and should be reinforced at all subsequent healthcare visits. Despite the high ED utilization of those at risk of suicide, however, studies have demonstrated that healthcare providers have been inconsistent in delivering LMR counseling to those at risk (Runyan et al. 2018; Betz et al. 2013). Therefore, the goal of this systematic review is to describe the scope of LMR counseling communicated by healthcare providers to individuals at risk of suicide, and to identify barriers to LMR counseling delivery in the ED.

## Methods

### Study eligibility

The protocol for this systematic review is registered with PROSPERO, the international prospective register of systematic reviews (CRD42018076734). Studies were selected using the PICOS framework for study inclusion. PICOS delineates study inclusion criteria by specifying parameters for populations, interventions, comparisons, outcomes, and study designs (Littell et al. 2008). Using this framework, inclusion criteria consisted of: (a) observational and experimental studies (b) studies that examined LMR counseling delivered in the ED regardless of patient age, sex or race/ethnicity, and (c) studies published in the English language. Exclusion criteria were as follows: (a) LMR practices other than counseling (e.g., online decision aid) (b) LMR counseling provided during inpatient care or hospitalization (c) LMR counseling provided outside of the healthcare setting, (d) conference presentations/proceedings.

### Data sources

The following databases were searched up to July 2020: (1) PUBMED, (2) Scopus, (3) PsycInfo, and (4) EBSCO. Search terms used to identify eligible studies included “suicide”, “self-harm”, “education”, “counseling”, “intervention”, “restriction”, “prevention”, “control”, “lethal means”, “LMR”, and “lethality”. A sample search strategy is provided in Table 1.

**Table 1:** Sample search strategy for study inclusion

[ TITLE-ABS-KEY [ sucid* OR self-harm ] ] AND [ [ [ lethal* OR toxic* ] ] AND [ education OR counseling OR intervention OR restriction OR reduction OR prevention ] ] AND [ control OR lmr OR lethal-means OR lethal-method ]	

### Study selection and data abstraction

Two researchers independently selected and coded all studies. Discrepancies were resolved by consensus of a third coder. Once eligible studies were identified, we searched for additional relevant studies in the reference lists of selected articles, and via inspection of existing article files. Results of individual studies were synthesized qualitatively. Data was abstracted into a codebook using Microsoft Excel. Variables of interest included characteristics of the study (e.g., author, year of publication, study period, location, recipient(s) of LMR counseling, lethal means addressed by counseling), study population, and attributes of LMR counseling delivery.

### Search precision

Number needed to read (NNR), an effort-to-yield measure of search precision, was calculated by dividing the number of included studies by the number of screened studies, after the removal of duplicates (Bachmann et al. 2002). The NNR is a valuable metric that allows researchers to estimate the number of studies that need to be screened before identifying one that meets the inclusion criteria, and is helpful in determining the necessary resources to replicate or conduct a similar study.

## Results

### Study characteristics

**Fig. 1:**
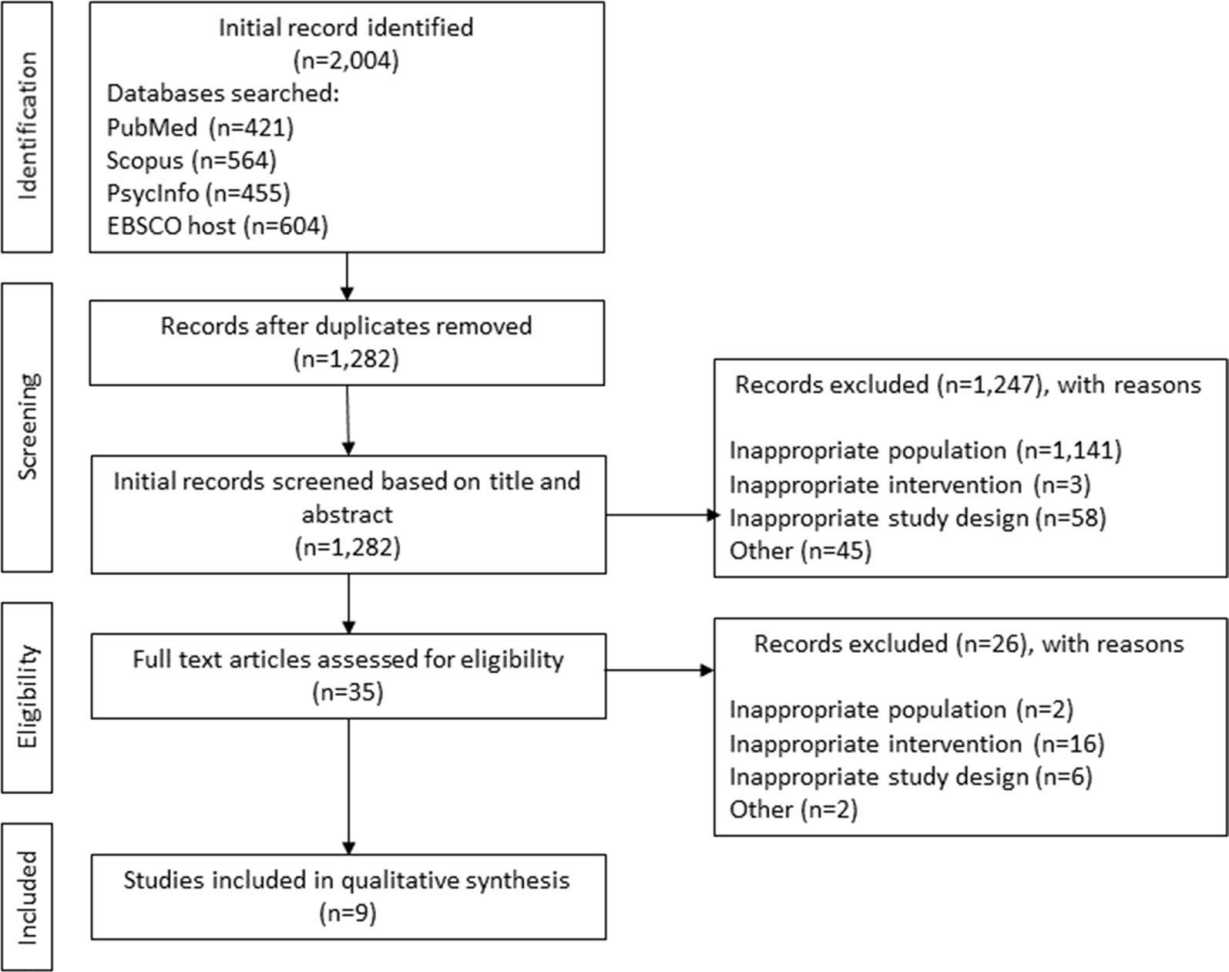
Flow diagram for the selection of studies

A total of 1282 studies were screened. Following a review of title and abstract, 35 full articles met the initial inclusion criteria, and 9 were included in this systematic review. Included studies were published from 1998 to 2020. Figure 1 describes the study selection process, including reasons for exclusion. Table 2 describes the characteristics of included studies. All studies were conducted in the United States; three were conducted in the West (Runyan et al. 2018, 2020; Bachmann et al. 2002) two were conducted in the Midwest (Fendrich et al. 1998; Kruesi et al. 1999), one was conducted in the Southeast (Sale et al. 2017) and three did not specify geographic location (Asarnow et al. 2017; Stanley and Brown 2012; Parast et al. 2018). Four studies classified the population density of the area in which they were conducted: two were conducted in urban settings (Fendrich et al. 1998; Parast et al. 2018) one was rural (Kruesi et al. 1999), and one encompassed both delineations (Runyan et al. 2018). Study designs included prospective follow-up & retrospective chart reviews (Kruesi et al. 1999; Runyan et al. 2016) a randomized controlled trial (Asarnow et al. 2017), a controlled trial (Runyan et al. 2020), a cross-sectional survey (Runyan et al. 2018), a cohort study (Fendrich et al. 1998), a quality improvement study (Parast et al. 2018), a pre/post-test survey (Sale et al. 2017), and a descriptive analysis of a safety planning intervention (Stanley and Brown 2012). The NNR was 142.

**Table 2:** Characteristics of included studies

References	Year	Type of delivery	Duration	Recipient of intervention	US region	Means discussed
Asarnow et al. (2017)	2017	Counseling by therapists in ED, home, and clinic	Multiple sessions	Parent/caregiver; patient	Not specified	Firearms, ligatures, medications, plastic bags, ropes, scarves, high places, places to hang from
Fendrich et al. (1998)	1998	Education by nurses & physicians in ED; focus on home firearm removal	Single session	Parent/caregiver	Midwest	Firearms
Kruesi et al. (1999)	1999	Education by ED staff during mental health assessment	Single session	Parent/caregiver	Midwest	Alcohol, firearms, medications [over-the-counter and prescribed]
Parast et al. (2018)	2018	Counseling by ED and inpatient staff	Single session	Parent/caregiver	Not specified	Car, firearms, medications
Runyan et al. (2018)	2018	Counseling by ED staff	Single session	Patient and family	West	Firearms and medications
Runyan et al. (2016)	2016	Counseling by behavioral health staff and physicians in ED; optional provision of lockbox	Single session	Parent/caregiver	West	Firearms and medications
Runyan et al. (2020)	2020	Counseling by behavioral health staff and physicians in ED; optional provision of lockbox	Single session	Parent/caregiver	West	Firearms and medications
Sale et al. (2017)	2017	Counseling by mental health providers	Single session	Patient and family	Southeast	Firearms, medicine, and chemical substances
Stanley and Brown (2012)	2012	Brief intervention including safety planning by clinician in ED	Single session	Patient	Not specified	Alcohol, firearms, knives, medications

### Patient characteristics

Included studies focused on a range of populations for LMR counseling. Studies that described the demographics of patients and/or caregivers receiving LMR counseling were majority female (Kruesi et al. 1999; Asarnow et al. 2017; Parast et al. 2018) and majority white (Kruesi et al. 1999; Parast et al. 2018). When indicated, the demographic characteristics of LMR counseling providers were also majority female (Fendrich et al. 1998; Sale et al. 2017) and majority white (Sale et al. 2017). LMR counseling targeted the parent/caregiver of a suicidal youth in five studies (Runyan et al. 2020, 2016; Fendrich et al. 1998; Kruesi et al. 1999; Parast et al. 2018), the parent/caregiver and the suicidal youth in one study (Asarnow et al. 2017), the adult suicidal patients and their families in two studies (Runyan et al. 2018; Sale et al. 2017) and only the patient themselves in one study (Stanley and Brown 2012).

### Delivery of LMR counseling

The implementation of LMR counseling also differed across studies. Counseling was provided during a single session in the ED in eight studies (Runyan et al. 2018, 2020, 2016; Fendrich et al. 1998; Kruesi et al. 1999; Sale et al. 2017; Stanley and Brown 2012; Parast et al. 2018). In addition to counseling within the ED, one study documented subsequent clinic and home visits over a three month period (Asarnow et al. 2017). The delivery of counseling was provided by mental health providers in three studies (Runyan et al. 2020; Sale et al. 2017; Asarnow et al. 2017) and by ED staff in six studies (Runyan et al. 2018, 2016; Fendrich et al. 1998; Kruesi et al. 1999; Stanley and Brown 2012; Parast et al. 2018).

### Scope of LMR counseling

Restricting access to firearms was the most common form of LMR counseling identified in included studies [n = 9], followed by limiting access to medications [n = 8] and alcohol [n = 2]. Other LMR counseling included restricting access to knives, chemical substances, and objects that could be used for asphyxiation such as ropes. Three studies mentioned offering locking devices or lock boxes to patients and/or caregivers (Runyan et al. 2020, 2016; Asarnow et al. 2017) and six did not (Runyan et al. 2018; Fendrich et al. 1998; Kruesi et al. 1999; Sale et al. 2017; Stanley and Brown 2012; Parast et al. 2018).

Few studies discussed the barriers to successful LMR counseling. Those that reported on barriers to implementation cited a lack of specific training and skills (Sale et al. 2017) and skepticism regarding its efficacy (Kruesi et al. 1999). Additionally, one study found that written protocols were significantly associated with increased LMR counseling on safe medication and firearm storage (Runyan et al. 2018).

## Discussion

Results of this systematic review suggests that there is great heterogeneity in the delivery of LMR counseling across EDs, supporting previous studies which have identified inconsistencies in LMR counseling delivery in healthcare settings (Runyan et al. 2018; Betz et al. 2013).

The ED serves as a critical point of contact for individuals at risk of suicide to receive immediate intervention, including referrals and/or services that address the underlying causes of anxiety, depression, hopelessness, and/or treatment for substance misuse. Despite evidence of the connection between mental health problems and suicidality, we identified just one study incorporating adherence to psychiatric treatment into LMR counseling (Stanley and Brown 2012). It has been shown that differing patterns of behavioral and emotional issues, which are highly informed by gender norms, may mediate the relationship between suicidal thoughts and suicidal behaviors (Peter and Roberts 2009). Women are more likely to suffer from internalizing disorders such as anxiety and mood disorders (Fergusson et al. 1993), but are also more likely to engage in help-seeking behaviors, to identify professionals and friends as sources of support, and have more culturally-sanctioned readiness to discuss emotional problems than do men (Beautrais 2002; Rickwood et al. 2005). In contrast, men more frequently suffer from externalizing disorders such as conduct or substance use disorders (Mergl et al. 2015), are more likely to engage in avoidance strategies (Gould et al. 2004), and are less likely to have been exposed to positive help-seeking behaviors (Rhodes et al. 2014). Therefore, an urgent priority should be to inform LMR counseling interventions using evidence-based gender-specific approaches to help-seeking.

Suicide has been shown to vary across a range of demographic characteristics. As such, we recommend that any approach to LMR counseling that is delivered in the ED be multifaceted and modifiable to the age, gender, and race-specific disparities in suicidality identified by extant literature. For example, the present study found that the majority of LMR counseling is delivered to women. This is interesting considering that men are the predominant victims of completed suicide. Our results may be explained by prior evidence suggesting that women are less likely to use lethal means, and more likely to seek medical care for suicidality than men (Beautrais 2002). According to the Centers for Disease Control and Prevention (CDC), males are more likely to attempt suicide using means that carry a high fatality rate (e.g., firearms, suffocation), while females are more likely to attempt suicide using poisoning (Leading Causes of Death Reports, 1981–2018 2020). Given the growing literature describing gender differences in suicide modality, LMR counseling should be flexible in order to appropriately address common means of suicide for men and women.

Similarly, the CDC has also reported disparities in suicide by race/ethnicity. Specifically, non-Hispanic black children under the age of 12 have a higher rate of suicide than their non-Hispanic white counterparts (Leading Causes of Death Reports, 1981–2018 2020). LMR counseling recommendations that are culturally diverse in content and delivery will be an important component to mitigating these racial differences.

Our search yielded just four studies in which LMR counseling was delivered directly to the patient. Excluding patients from this important discussion may be a missed opportunity for safety around suicidal triggers and access which may prove lifesaving. It is imperative that suicidal patients be active participants in LMR counseling, so that efforts to mitigate lethality can be customized according to individual risk factors and access.

### Limitations

This study is not without limitations. First, the reliability and accuracy of the results and methods reported in each of the nine included studies contribute to the overall reliability of this systematic review. Second, included studies were selected based upon a predetermined list of terms and phrases related to suicide and LMR. Because LMR is a fairly recent concept, relevant literature may not have been identified due to unidentified variations in terminology. Finally, some relevant patient and visit level characteristics were missing from the included studies [e.g., age of patients, didactic methods of delivery], that have the potential to contextualize the utility of LMR counseling. We recommend that these characteristics be included in future studies.

## Conclusion

There is limited evidence identifying the most effective methods and target populations for LMR counseling. Given the growing literature providing evidence of gender differences in suicide modality (e.g., guns, medications, suffocation), lethal means restriction education should address common means of suicide based on age and gender. A majority of suicide attempts and half of all completed suicides do not involve firearms (Leading Causes of Death Reports, 1981–2018 2020), regardless of age or gender. This highlights the need to include lethal means counseling that addresses multiple suicide modalities, to reduce risk of suicide. Further prospective studies should identify and evaluate the most effective method(s) of providing lethal means counseling.

## Data Availability

Not applicable.

## Abbreviations

LMR: Lethal means restriction
ED: Emergency department
